# Blindness and Glaucoma: A Multicenter Data Review from 7 Academic Eye Clinics

**DOI:** 10.1371/journal.pone.0136632

**Published:** 2015-08-24

**Authors:** Luca Rossetti, Maurizio Digiuni, Montesano Giovanni, Marco Centofanti, Antonio M. Fea, Michele Iester, Paolo Frezzotti, Michele Figus, Antonio Ferreras, Francesco Oddone, Lucia Tanga, Teresa Rolle, Valentina Battaglino, Chiara Posarelli, Ilaria Motolese, Pietro Mittica, Simone Alex Bagaglia, Cristina Menicacci, Stefano De Cilla’, Alessandro Autelitano, Paolo Fogagnolo

**Affiliations:** 1 Dipartimento Testa-Collo, San Paolo Hospital, University of Milan, Milan, Italy; 2 G. B. Bietti Foundation—IRCCS, Rome, Italy; 3 Università degli Studi di Torino, Turin, Italy; 4 Clinica Oculistica, Università di Genova, Genoa, Italy; 5 Dipartimento di Scienze oftalmologiche e Neurochirurgiche, Universita' degli Studi di Siena, Siena, Italy; 6 Eye Clinic, Department of Neuroscience, University of Pisa, Pisa, Italy; 7 Department of Ophthalmology, Miguel Servet University Hospital, University of Zaragoza, Zaragoza, Spain; 8 Unit of Ophthalmology, Ospedale Maggiore della Carità, Novara, Italy; Harvard Medical School, UNITED STATES

## Abstract

**Purpose:**

To evaluate frequency, conversion rate, and risk factors for blindness in glaucoma patients treated in European Universities.

**Methods:**

This multicenter retrospective study included 2402 consecutive patients with glaucoma in at least one eye. Medical charts were inspected and patients were divided into those blind and the remainder (‘controls’). Blindness was defined as visual acuity≤0.05 and/or visual field loss to less than 10°.

**Results:**

Unilateral and bilateral blindness were respectively 11.0% and 1.6% at the beginning, and 15.5% and 3.6% at the end of the observation period (7.5±5.5 years, range:1–25 years); conversion to blindness (at least unilateral) was 1.1%/year. 134 eyes (97 patients) developed blindness by POAG during the study. At the first access to study centre, they had mean deviation (MD) of -17.1±8.3 dB and treated intraocular pressure (IOP) of 17.1±6.6 mmHg. During follow-up the IOP decreased by 14% in these eyes but MD deteriorated by 1.1±3.5 dB/year, which was 5-fold higher than controls (0.2±1.6 dB/year). In a multivariate model, the best predictors for blindness by glaucoma were initial MD (p<0.001), initial IOP (p<0.001), older age at the beginning of follow-up (p<0.001), whereas final IOP was found to be protective (p<0.05).

**Conclusions:**

In this series of patients, blindness occurred in about 20%. Blindness by glaucoma had 2 characteristics: late diagnosis and/or late referral, and progression of the disease despite in most cases IOP was within the range of normality and target IOP was achieved; it could be predicted by high initial MD, high initial IOP, and old age.

## Introduction

The socio-economic impact of glaucoma is very high mainly due to its epidemiology and the effects on visual function. The disease has a high prevalence (> 66 million people worldwide) and is the second leading cause of irreversible blindness (>7 million people bilaterally blind worldwide) [1]. A recent review found that glaucoma is responsible for 10–11% of blindness in Western Europe and U.S., and this percentage is increasing in the last decade [2]. Progressive glaucomatous visual field (VF) impairment is associated with reduced vision-related quality of life [3,4] and higher social costs [5].

The visual outcome is the major concern of glaucoma patients [6]. At diagnosis, 34% are worried about the probability of becoming blind in the future; even if this percentage decreases to 11% at follow-up, fear is still very high for patients with severe field deterioration and progression [7].

The topic of blindness in glaucoma is still controversial despite its relevance. Prospective studies provided estimates for blindness due to primary open-angle glaucoma (POAG) in at least one eye of 15–27%, and in both eyes of 6–12%, with incidence of about 1% per year (follow-up ranging from 15 to 34 years) [8–11], but other studies suggested that blindness incidence is declining due to the advent of modern treatments [12,13]. A recent study from Malmo (Sweden) on a cohort of about 600 patients followed from diagnosis to death found a prevalence of 42% of blindness in one eye and 16% in both eyes at the last visit [14]. A Chinese study on primary angle-closure glaucoma (PACG) estimated blindness at presentation as 6% and 30.1% based on visual acuity (VA) and visual field (VF) criteria with a progression to blindness in 7% over a 10-year follow-up [15]. Large differences can be due to race [16] and, moreover, the several definitions of blindness may raise confusion [17,18]. Most of these studies were conducted on small samples of selected patients with exclusion of ocular comorbidities, so that a clear scenario of the prevalence and causes of blindness in glaucoma practice is still only partially depicted.

The aim of this study is to report on the frequency, conversion, causes and risk factors of blindness in a large cohort of glaucoma patients from different academic centers.

## Materials and Methods

This retrospective data review involved 7 European sites: the University Eye Clinics of Milan (San Paolo Hospital), Turin, Genoa, Pisa, Siena, Rome (Tor Vergata)–Italy, and the Department of Ophthalmology, Miguel Servet Hospital, University of Zaragoza, Zaragoza–Spain. The study was approved by the Ethics Committee at each study site, and respected the tenets of the Declaration of Helsinki and national laws for the protection of personal data. Written informed consent was obtained from all the participants.

Each of the seven centres included 350 patients with a documented diagnosis of POAG in at least one eye; excluded were patients with age <18 years and cases of ocular hypertension or glaucoma suspect. Patients’ data were retrieved between January and June 2010 from the Hospital databases in alphabetical order; in case of families, only the first patient in the database was included. No selection was done based on patients provenance (included were both referred and non-referred patients, at any stage of severity of the disease), VF, optic nerve head, intraocular pressure (IOP) or VA.

A diagnosis of glaucoma was accepted if at least one eye had a repeatable VF change consistent with glaucoma together with a glaucomatous optic nerve head appearance (manifest glaucoma). Patients with POAG had to have an open-angle and no secondary cause of IOP elevation. For the purposes of the study, blindness had to be attributed to a single pathology between POAG, PACG, macular degeneration, high myopia, vein occlusion, trauma, retinal detachment, diabetes, and corneal pathologies. The cause of blindness was determined by reviewing the patient charts and through analysis of the VFs; in presence of comorbidity, consensus between investigators was reached.

The World Health Organisation (WHO) definition of blindness was used [14,19]: best-corrected VA of less than 0.05 and/or VF loss to less than 10°. Blindness criteria were confirmed by at least two consecutive visits. Patients with reversible blindness (due to perimetric variability, cataract surgery, or glaucoma course itself; see Results section) were not analyzed as blind. Perimetries were performed at all sites using Humphrey Field Analyzer 750 (Carl Zeiss Meditec, Dublin, CA, USA), full-threshold or SITA Standard programs over the central 30 or 24 degrees, using III spot size.

For each included patient we recorded age, sex, medical history (cardiovascular factors included diabetes, carotid stenosis cardiac and vascular diseases except Horton disease), any eye surgical procedures, number of visits, procedures performed at each visit, an anterior and posterior segment description, VF, VA, IOP values, type of ocular treatment, adherence to treatment and referral to study center, as derived from medical charts. Time-points were: date of diagnosis (when a glaucomatous VF defect together with an optic nerve head damage was first shown), date of the first visit at the study center (beginning of follow-up), date of occurrence of blindness (if this was the case), date of the last visit (end of follow-up).

### Statistical analysis

The whole sample was analyzed to assess the global frequency (or prevalence), conversion rate (or incidence) and the different causes of blindness. The risk of conversion to blindness was evaluated only among patients diagnosed with POAG. To account for different follow up times and time varying covariates, a parametric accelerated failure model (Weibull distribution) was used. Model included stratification by center to account for between-center variability. For each patient, blindness from glaucoma of at least one eye occurring during the time of observation was considered as an event (failure). Since the exact time of occurrence of blindness was not available, interval censoring was used. For covariates that implied one observation from each eye (i.e. Mean Deviation, MD, and IOP), the mean of the two values was calculated for each patient. Finally, a standard linear model was used to investigate the final MD and its correlation with different covariates. In this case, each eye was considered individually and correlation between observations was corrected with the addition of random effects (for both subject and center factors). Different number of cases and observations among fitted models are due to missing values for the different covariates considered in each case. Models were modified to avoid obvious multicollinearity among predictors (e.g. the final IOP and the initial IOP were never in the same model, see Results). All calculations were performed in R scripting environment [20–23].

## Results

A total of 2,402 patients were included in the dataset; race was Caucasian in 99%, other in 1% (African n = 18, Hispanic n = 6, Indian n = 3); 99% of patients were literate; 55% were female. In 52 cases, blindness at the first visit was not confirmed at follow-up: 27 eyes had VA improvement thanks to cataract surgery; 6 had sudden IOP reduction (of these 6 referred patients, 4 received trabeculectomy, and 2 trabeculoplasty and maximum-tolerated medical treatment) and were not confirmed as blind due to VF; finally, 19 patients showed perimetric learning effect. All of these subjects were included in the analysis as non-blind patients.

Mean age of the study population at the beginning of follow-up was 68.7±11.5 years, with a statistically significant difference between non-blind (66.8±11.8 years) and blind patients (72.0±10.1 years, P<0.0001). Mean follow-up was 7.5±5.5 years (median 7, range 1–25 years); for non-blind patients it was 7.3±5.4 years (median 7, range 1–24 years), for blind patients 8.4±6.0 years (median 8, range 1–25 years, P = 0.047). Blindness occurred at 73.3±10.2 years; 70% of the totality of blind cases occurred before referral to the study centers; 51% of blind patients were female.

At the beginning of follow-up, the frequency of unilateral blindness was 11.0% (262 eyes; 262 patients), whereas bilateral blindness was present in 1.6% (39 patients). At the end of the study, the frequency of unilateral and bilateral blindness respectively increased to 15.5% (372 eyes of 372 patients) and 3.6% (86 patients).

The number of eyes converted to blindness in the course of the study was 204 of 157 patients. In these patients, the mean time elapsed from first observation to blindness was 4.6±3.0 years (range: 1–21 years). Conversion to blindness (at least unilateral) was 1.1% per year. Frequency of blindness was similar between centres (p>0.20, Weibull test).

The causes of blindness are reported in Table 1. As expected, POAG was the main cause (61.4%), whereas PACG accounted for 7.2% of cases. Glaucoma patients frequently had other eye diseases determining blindness: macular degeneration (7.6%), high myopia (5.7%), vein occlusion (4.8%), trauma (3.5%), retinal detachment (3.1%), diabetes (1.5%), and corneal pathologies (1.3%). When the analysis was restricted only to eyes that converted to blindness during the study, similar figures were found with the exception of a lower prevalence of trauma, retinal detachment and corneal pathologies.

**Table 1:** Causes of blindness in the study.

Causes	% of blind eyes at the end of the study (n = 544)	% of eyes converted to blindness during the study (n = 204)
** POAG**	61.4	65.6
** PACG**	7.2	5.7
** ARMD**	7.6	7.6
** Myopia**	5.7	7.6
** Vascular diseases (BVO, CVO)**	4.8	7.6
** Trauma**	3.5	1.3
** Retinal detachment**	3.1	1.3
** Diabetic retinopathy**	1.5	1.9
** Cornea**	1.3	0.6
** Other**	3.9	0.6

ARMD, age-related macular disease; BVO, branch vein occlusion; CVO, central vein occlusion; PACG, primary angle-closure glaucoma; POAG, primary open-angle glaucoma.

Further analysis was conducted on eyes blind due to POAG (n = 334). 60% (n = 200) were blind at first observation, whereas 40% (n = 134) developed blindness due to POAG during the study period. For the 200 eyes of patients blind by POAG at the beginning of the study, the criterion of blindness was VF in 93%, VA in 12%, and both in 5%. These patients were referred to the study centers more frequently than non-blind cases (53% vs 40%, P<0.001) and had MD of -26.5±4.8 dB at first examination.

The 134 eyes who developed blindness by POAG in the course of the study had MD of -17.1±8.3 dB (median -17 dB) and IOP of 17.1±6.6 mm Hg (median 17 mmHg) at presentation (Table 2). The main feature of these patients is that their fields deteriorated regardless of aggressive IOP treatment: all patients received maximum-tolerated medical treatment and, in 75% of cases, glaucoma laser or surgery; this determined a further mean IOP decrease to 14.6±3.5 mm Hg (median 14 mmHg, -14%).

**Table 2:** Characteristics of IOP in patients developing blindness by POAG in the course of the study.

	Beginning of the study	End of the study
**Mean IOP (mmHg)**	17.1 ± 6.6	14.6 ± 3.5
**Range (mmHg)**	8–56	6–36
**IOP > 18 mm Hg (%)**	17%	9%

IOP, intraocular pressure (mean of the two eyes).

In these 134 eyes, the rate of MD change was -1.1±3.5 dB / year, which was 5-fold higher than non-blind subjects (-0.2±1.6 dB / year). As expected, some comorbidities were associated with increased rate of progression: the rate of change was -1.8±1.1 dB / year in presence of macular degeneration, and -3.1±1.4 dB / year in presence of vein occlusion. Myopia did not significantly modify the rate of perimetric progression, apart in 3 eyes having a mean change of -1.9 dB / year.

Next, analysis of conversion rate was performed on patients who had at least one eye at risk at the beginning of the study (i.e. not blind) and whose conversion could not be attributed to different causes other than POAG and for whom complete data were available. In this analysis 1606 patients were analyzed and 53 converted to blindness from at least one eye by the end of follow up. Of the covariates analyzed in the survival model for blindness, only age at the beginning of the follow up, initial MD value and final IOP have shown to be significant predictors. Table 3 shows the same multivariate survival model fitted with the Initial IOP or the Final IOP as predictors, together with mean Initial MD and Age. Since all calculations were made to estimate the probability of becoming blind from at least one eye, MD and IOP value were calculated for each subject as the mean of the values from the affected eyes. As expected, the major risk factor was the severe initial MD (p<0.0001), which can be thought as an indicator of the stage of the disease at the beginning of the follow up. Higher age at the beginning of the study was a significant risk factor for blindness (p<0.0001). Final IOP interestingly showed a slightly protective effect (p = 0.008) meaning that eyes with lower pressure values at the end of the study were more likely to become blind.

**Table 3:** Multivariate survival model fitted with the Initial IOP (first column) or the Final IOP (second column) as predictors, together with mean Initial MD and Age.

	Blindness, Weibull parameter estimates (standard error)	
**Age**	-0.029 (0.013)**	-0.027 (0.013)**
**Mean MD**	0.105 (0.023)***	0.110 (0.024)***
**Mean Final IOP**	0.090 (0.036)**	-
**Mean Initial IOP**	-	0.016 (0.028)
**Intercept**	6.987 (1.212)***	7.996 (1.357)***
**Observations**	1,606	1,606

*p<0.1

**p<0.05

***p<0.01

IOP, intraocular pressure; MD, Mean Deviation; Initial, at the beginning of follow up; Final, at the end of follow up.

Since all calculations were made to estimate the probability of becoming blind from at least one eye, MD and IOP value were calculated for each subject as the mean of the values from the affected eyes.

Table 4 shows the hazard ratios for each risk factor. Since the model used is a parametric accelerated failure model with a Weibull distribution, the hazard ratio depends on the scale parameter, which is different for each center due to stratification. The presented hazard ratios are a global calculation (i.e. with no strata) although the significance of the covariates was assessed with a stratified model. Similar results could be found by analyzing the correlation between the same variables and the MD at the end of the follow up.

**Table 4:** Hazard ratios and confidence intervals for each risk factor in determining blindness.

	HR	Lower 95% CI	Upper 95% CI
**Age**	1.0321506	1.0082546	1.0566128
**Mean MD**	0.8962068	0.8690180	0.9242462
**Mean Final IOP**	0.9040504	0.8394736	0.9735948

IOP, intraocular pressure; MD, Mean Deviation; Final, at the end of follow up. Since the model used is a parametric accelerated failure model with a Weibull distribution, the hazard ratio depends on the scale parameter, which is different for each center due to stratification. The presented hazard ratios are a global calculation (i.e. with no strata) although the significance of the covariates was assessed with a stratified model.

Tables 5 and 6 show the same multivariate linear model fitted with Final MD values as dependent variable and Initial IOP (Table 5) or Final IOP (Table 6) as predictors, together with other predictors. Table 7 shows a multivariate regression of the Initial MD value on the other predictors in Table 5. Again, the initial MD value was the best predictor of the final MD (p<0.0001, correlation coefficient 0.9). Besides the obvious correlation with the follow up time, other important factors were age (p<0.0001) at the beginning of the follow up and surprisingly the initial IOP (p<0.0001) but not the final IOP. This latter apparent discrepancy shows that the final IOP had indeed little role in the actual progression of the visual field impairment, besides the efforts taken to lower the pressure levels in these patients. On the other hand, the significant correlation with the initial IOP can be better explained considering its role in determining the initial MD value (Table 7).

**Table 5:** Table shows a multivariate linear model fitted with Final MD values as dependent variable and Initial IOP as predictor, together with other predictors.

	Final MD, Estimated parameters (standard errors)
**Cardiovascular**	-0.238 (0.229)
**Hypertension**	0.104 (0.185)
**Hypotension**	-0.318 (0.500)
**Non Family History of Glaucoma**	-0.311 (0.194)
**Initial MD**	0.841 (0.012)***
**Age**	-0.043 (0.007)***
**Follow up time**	-0.138 (0.025)***
**Initial IOP**	-0.061 (0.016)***
**Intercept**	3.492 (0.589)**
**Observations**	3,246
**Log Likelihood**	-9,076.319

*p<0.1

**p<0.05

***p<0.01

IOP, intraocular pressure; MD, mean deviation

**Table 6:** Multivariate linear model fitted with Final MD values as dependent variable and Final IOP as predictor, together with the same predictors as in Table 5. (except for the Initial IOP).

	Final MD, Estimated parameters (standard errors)
**Cardiovascular**	-0.236 (0.230)
**Hypertension**	0.115 (0.185)
**Hypotension**	-0.290 (0.501)
**Non Family History of Glaucoma**	-0.289 (0.195)
**Initial MD**	0.843 (0.012) ***
**Age**	-0.041 (0.007) ***
**Follow up time**	-0.140 (0.025) ***
**Final IOP**	-0.009 (0.024)
**Intercept**	2.401 (0.638) **
**Observations**	3,246
**Log Likelihood**	-9,082.704

*p<0.1

**p<0.05

***p<0.01

IOP, intraocular pressure; MD, mean deviation

**Table 7:** Multivariate regression of the initial Mean Deviation on the other predictors of Table 5.

	Initial Mean Deviation, Estimated parameters (standard errors)
**Cardiovascular**	-0.991 (0.374) ***
**Hypertension**	0.315 (0.303)
**Hypotension**	-1.200 (0.821)
**Non Family History of Glaucoma**	-0.377 (0.318)
**Age**	-0.100 (0.011)***
**Initial Intraocular Pressure**	-0.065 (0.026)**
**Intercept**	2.306 (0.922) **
**Observations**	3,246
**Log Likelihood**	-10,386.370

*p<0.1

**p<0.05

***p<0.01

Initial MD was also strongly correlated with the presence of cardiovascular factors (which included diabetes, carotid stenosis and cardiac diseases). However, multicollinearity of predictors (as evident from Table 7) can limit the validity and consequently the interpretation of these models.

## Discussion

This paper explored the epidemiology and causes of blindness in a group of patients followed by the “Glaucoma Services” of 7 academic centers. Unilateral and bilateral blindness was respectively 11.0% and 1.6% at the beginning of the study, and 15.5% and 3.6% at the end of the observation period (7.5±5.5 years), with a conversion of 11 new blind patients/1,000 per year.

Overall, these findings are consistent with those from prospective studies [8–11]. In our dataset, causes of blindness other than POAG and PACG accounted for about 30% of cases (Table 1). The WHO criteria used in this study are by far more restrictive than those for legal blindness (VA < 0.1 and/or VF constriction within central 20°) used in other studies [8–13]. WHO criteria, to us, address the concept of “blindness” better than legal criteria: most of our patients with VF constriction at 20° can lead a normal life, apart from driving restriction, and in the US it is estimated that 90% of “legally blind” subjects have residual vision [16].

As confirmed by previous studies [10,17], blind patients were older than controls; no significant sex difference was found. In glaucoma patients developing blindness during the study, blindness was associated with late detection of the disease [24,25], advanced VF damage at diagnosis [11,26–28]; these patients were referred to the study centers more frequently than controls [24].

IOP is considered a major risk factor for progression to blindness in glaucoma [11,28–32]. In our study patients going blind progressed despite IOPs in the ‘normal’ range (17.1±6.6 mm Hg–values very close to Chen’s study [8]). In this group, some patients had very high IOP values (maximum IOP was 56 mm Hg), so that, if these outliers had been excluded, mean IOP would have been even lower. During the study, further IOP reduction of 14% was obtained, but this did not halt VF progression, in contrast with the data of the Canadian Glaucoma Study [33]. Possible explanations include the different amount of IOP reduction in the 2 studies and the fact that, differently from our study, in the Canadian Glaucoma Study a target IOP was searched in all progressing patients. On the other side, we confirmed the role of sudden IOP reduction in recovering visual function, at least on a minority of patients [34–36].

These results clearly show that one of the major determinants of blindness and disease progression in glaucoma is the initial condition of the patients upon referral to second level centers. Although this might seem obvious, it points out that correct timing in diagnosis and treatment is fundamental to properly control visual field loss and prevent blindness. It is important to notice that in this study, known important factors, such as IOP and cardiovascular factors, were only significant in determining the initial MD value. This could either depend on the fact that the follow up period was not sufficient to properly evaluate the effect of such variables or to the fact that proper timing and treatment can effectively control the progression of the disease in spite of these differences among patients. Of course, this analysis suffers from the fact that only few patients (slightly more that 2% of the considered eyes) were blind at the end of the study. Nevertheless, these conclusions are also supported by the analysis on the progression of visual field loss which eliminates any distortion introduced by the “threshold” effect intrinsic to the definition of blindness.

The initial IOP could be considered as an indicator of pressure control before entering the study and thus important in setting the initial visual field condition and, secondly, the final MD.

Higher final IOP was found to be protective for blindness. This paradox could be explained considering that patients at risk of becoming blind in a short time (typically patients with advanced glaucoma) usually receive aggressive IOP-lowering treatments, although this is frequently insufficient to prevent blindness. It is therefore evident that final IOP may have a small role in the actual progression of VF damage, despite the efforts taken to lower the pressure levels in these patients.

We explored the effect of age at the beginning of follow up and our results matched the findings of other reports [14,37], showing that older patients are more susceptible to visual impairment and blindness by glaucoma. It is unlikely that these differences could arise from different disease durations since the initial MD values considered in the multivariate analysis served also as a control factor for the initial patient conditions.

Cardiovascular factors were found to be significantly correlated with more negative initial MD values but not with final MD values and the risk of blindness. This could be explained by the fact that cardiovascular factors are more important on long time periods, thus setting the initial severity of visual field impairment, but are not crucial on a smaller time scale, i.e. the duration of follow up. Caution is necessary when evaluating these data, as cardiovascular factors (as well as hypertension and hypotension anamnestic data) were self reported by patients regardless of chronic treatments and control of the diseases.

Our study has some strengths and limitations. The strengths include the large dataset, the absence of any patient selection, and the data consistency between centers. Moreover, we used the stringent criteria for blindness suggested by WHO and we confirmed blindness frequencies given by Forsman [9] and Oliver [11]. On the other hand, our results may be limited by the retrospective design of the study and the fact that the quality of the analysis relies on the quality of the medical charts. This is particularly relevant for the study of risk factors, which was based exclusively on the medical history referred by patients. Also, we preferred not to divide POAG from pseudoexfoliative, pigmentary, and normotensive glaucoma, even if these glaucomas have different prognosis [38]. In fact, a precise diagnosis could not be done in many cases (absence of or inadequate baseline data in referred patients; cataract surgery, which could alter a correct assessment of pseudoexfoliation and pigmentary glaucoma). In such a study, it is also hard to separate the effects of cataract on the progression of VF mean deviation.

The principal limitation of this study is that patient catchment of the academic centres is unlikely to represent the patient catchment of the hospital system as a whole. In fact, study centres have their own catchment from the community (i.e. patients requiring an ophthalmic examination have unrestricted access to a primary eye care service), but also a significant number of tertiary referrals (i.e. patients seen in other hospitals for various reasons, including the need for assistance with management of severe glaucoma). It should be noted that the prevalence of blindness by glaucoma in our study is similar to literature [8–11]; we therefore assume that the effect of a selection bias, if present, is not large. Yet, in our study, 70% of all blind cases occurred before referral to the study centres. Strategies of data capture across hospital systems as a whole are recommendable in order to depict a clear scenario of the burden of blindness associated with glaucoma in hospital care; the progressive use of digitized data and telemedicine will be helpful in this process.

To summarize, this paper has the merit of showing that glaucoma is still a very dangerous disease, as about 20% of glaucoma patients followed in University Eye Clinics are blind in at least one eye. Glaucomas caused 70% of blindness, and two features could be identified for these patients: late diagnosis and/or late referral, and progression of the disease despite “generally accepted” IOP values. Patients going blind had a decrease of mean deviation of about 1 dB/year, which is very similar to the natural history of untreated Caucasian glaucoma patients [39], and five-fold higher than non-blind glaucoma patients.

Screening strategies for glaucoma have been shown to be inadequate in view of the ratio between costs and effectiveness. Still, the problems of late diagnosis and of the high frequency of blindness remarked by this paper highlight the inadequacy of the current strategies for glaucoma diagnosis, even in 2012 in so-called “developed” Countries.

Finally, we showed that people going blind progress more rapidly than controls and that, in advanced stages, progression seems to become less affected by IOP reduction. We therefore recommend to assess risk factors, to measure VF progression and to provide aggressive treatments in patients showing high progression (loss of mean deviation>1dB/year) at earlier stages, in order to reduce the likelihood of developing blindness.

## Supporting Information

S1 File Datasheet of risk factors. (CSV)

S2 File Datasheet of multivariate analysis (final mean deviation and final intraocular pressure). (CSV)

S3 File Datasheet of multivariate analysis (initial mean deviation and other predictors). (CSV)
