# Correction: Blindness and Glaucoma: A Multicenter Data Review from 7 Academic Eye Clinics

**DOI:** 10.1371/journal.pone.0151010

**Published:** 2016-03-02

**Authors:** Luca Rossetti, Maurizio Digiuni, Giovanni Montesano, Marco Centofanti, Antonio M. Fea, Michele Iester, Paolo Frezzotti, Michele Figus, Antonio Ferreras, Francesco Oddone, Lucia Tanga, Teresa Rolle, Valentina Battaglino, Chiara Posarelli, Ilaria Motolese, Pietro Mittica, Simone Alex Bagaglia, Cristina Menicacci, Stefano De Cilla’, Alessandro Autelitano, Paolo Fogagnolo

The third author’s name is spelled incorrectly. The correct name should be: Giovanni Montesano. The correct citation should be: Rossetti L, Digiuni M, Montesano G, Centofanti M, Fea AM, Iester M, et al. (2015) Blindness and Glaucoma: A Multicenter Data Review from 7 Academic Eye Clinics. PLoS ONE 10(8): e0136632. doi:10.1371/journal.pone.0136632
